# Effects of rainfall patterns and land cover on the subsurface flow generation of sloping Ferralsols in southern China

**DOI:** 10.1371/journal.pone.0182706

**Published:** 2017-08-08

**Authors:** Jian Duan, Jie Yang, Chongjun Tang, Lihua Chen, Yaojun Liu, Lingyun Wang

**Affiliations:** 1 School of Soil and Water Conservation, Beijing Forestry University, Beijing, China; 2 Jiangxi Institute of Soil and Water Conservation, Nanchang, China; RMIT University, AUSTRALIA

## Abstract

Rainfall patterns and land cover are two important factors that affect the runoff generation process. To determine the surface and subsurface flows associated with different rainfall patterns on sloping Ferralsols under different land cover types, observational data related to surface and subsurface flows from 5 m × 15 m plots were collected from 2010 to 2012. The experiment was conducted to assess three land cover types (grass, litter cover and bare land) in the Jiangxi Provincial Soil and Water Conservation Ecological Park. During the study period, 114 natural rainfall events produced subsurface flow and were divided into four groups using k-means clustering according to rainfall duration, rainfall depth and maximum 30-min rainfall intensity. The results showed that the total runoff and surface flow values were highest for bare land under all four rainfall patterns and lowest for the covered plots. However, covered plots generated higher subsurface flow values than bare land. Moreover, the surface and subsurface flows associated with the three land cover types differed significantly under different rainfall patterns. Rainfall patterns with low intensities and long durations created more subsurface flow in the grass and litter cover types, whereas rainfall patterns with high intensities and short durations resulted in greater surface flow over bare land. Rainfall pattern I had the highest surface and subsurface flow values for the grass cover and litter cover types. The highest surface flow value and lowest subsurface flow value for bare land occurred under rainfall pattern IV. Rainfall pattern II generated the highest subsurface flow value for bare land. Therefore, grass or litter cover are able to convert more surface flow into subsurface flow under different rainfall patterns. The rainfall patterns studied had greater effects on subsurface flow than on total runoff and surface flow for covered surfaces, as well as a greater effect on surface flows associated with bare land.

## Introduction

Water is the primary cause of soil erosion in southern China, a region known for the production of tropical crops and grains [1]. For a long time, soil and water losses have been the primary sources of soil degradation in southern China, resulting from traditional farming practices and the unsustainable utilization of slope lands. In recent years, the improper regulation of large-scale farmlands and orchards has become a major cause of erosion on Ferralsol slopes, mainly because of the shift in agriculture and forestry to an industrial structure [2, 3]. New technology must be urgently developed to improve the synergy between agricultural production and ecological functions.

Soil erosion due to surface runoff plays an important role in land degradation and desertification [4, 5]. Land cover and rainfall patterns are the two main factors that affect the intensity and frequency of runoff generation [6–10]. Understanding the effects of rainfall patterns and surface cover on runoff production will support soil and water conservation [11]. Surface cover is an important indicator of soil and water conservation and is widely used to prevent soil and water losses on sloped lands [12–14]. Grass cover and litter cover are the two most frequently used forms of surface cover. Land cover, including living vegetation and litter, can significantly reduce the volume of surface runoff by increasing water infiltration into the soil [13, 15, 16]. In addition, rainfall patterns have an important effect on the rainfall-surface runoff process on the Loess Plateau [8], and research in the Ferralsol region has produced similar results [9, 10]. The impacts of land cover and rainfall patterns on rainfall-surface runoff processes have received considerable attention. However, less attention has been paid to determining the effects of rainfall patterns and surface cover on subsurface flow generation. Fu et al. [17] and Xie et al. [18] concluded that rainfall intensity and quantity were the primary factors affecting subsurface flow generation based on rainfall simulations and simple, natural rainfall events.

Subsurface flow is also an important rainfall-runoff component that influences soil and water conservation [11, 19, 20]. Selecting the best method to quantify subsurface flow and determining the associated generation mechanisms are important and difficult problems in the field of soil hydrology research. Two major investigation methods are used to research subsurface flows at large scales: trench investigations [21] and isotope tracing [22]. Dye tracers [23, 24] and ground penetrating radar [25] have been used in rainfall simulation experiments at the plot scale. The results of these simulations and runoff plot observations have improved our understanding of the mechanisms of subsurface flow generation. However, long-term in situ monitoring data have yet to fully represent subsurface flows.

The long-term impacts of mechanical tillage and vegetation litter (root) have resulted in loose, highly permeable soils in the shallow layer of sloping Ferralsols and compact, low-permeability soils in the deep layer. Subsurface flows are most likely generated by water infiltration into the deep layer [26–28]. Subsurface flow discharges can exceed those of surface runoff and become the primary rainfall-runoff component during rainfall events [18]. The unique seasonal characteristics of a subtropical humid climate and the specific vertical profile properties of Ferralsols are associated with different characteristics and mechanisms of subsurface flows relative to those in purple soil areas and karst regions [17, 26].

In the present study, grass cover, litter cover and bare land runoff plots were established to collect in situ measurements of surface and subsurface flows in areas with sloping Ferralsols under natural rainfall conditions in southern China. Overall, 114 rainfall events from 2010 to 2012 were classified into four rainfall patterns using the k-means clustering method according to rainfall depth, maximum 30-min intensity and rainfall duration. The main objectives of this study are as follows: (1) to analyze the effects of different land cover types on surface and subsurface flows under natural rainfall conditions using in situ field observations, (2) to determine the responses of surface and subsurface flows to different rainfall patterns, and (3) to understand the roles of different land cover types in the proportions of surface and subsurface flows for different rainfall patterns.

## Materials and methods

### Ethics statement

The runoff plots in this study were managed by Jiangxi Provincial Soil and Water Conservation Ecological Park, a state-owned Science Park in Jiangxi province, China. Our study complies with the current laws of China and international rules, and no other specific permissions were required for these activities. In addition, the field study did not involve endangered or protected species.

### Study area

The data in this study were collected between 2010 and 2012 in the Jiangxi Provincial Soil and Water Conservation Ecological Park in the Yangou watershed of De’an County (115°42'38″-115°43'06″E, 29°16'37″-29°17'40″N), which is located in the hilly Ferralsol region of southern China. The experimental area is dominated by a subtropical and humid climate with a mean annual precipitation of 1469 mm. Seasonal precipitation in the area is concentrated between April and September. Mean annual air temperature is 16.7°C; annual sunshine duration ranges from 1700 to 2100 h; the frost-free period ranges from 245 to 260 days; and elevation ranges from 30 to 90 m.

The Ferralsols in this region are predominantly weathered from Quaternary sediments. The soil depth in the study area is over 100 cm, and the profile is characterized as “Ah-Bs-Cs”. The Ah layer depth varies between 25 and 30 cm, with a soil bulk density of 1.05–1.32 g·cm^-3^. The soil structure of the Ah layer is more porous than that of the Bs layer, which has a mean soil bulk density of 1.48 g·cm^-3^ from a depth of 30–60 cm. In this area, the vegetation is characterized by an evergreen broad-leafed forest (EBLF). The existing vegetation consists of a secondary community in different stages of recessive succession. The artificial and restored native shrub and tree community is primarily dominated by conifers.

### Experimental plot set up and in situ observations

**Fig 1:**
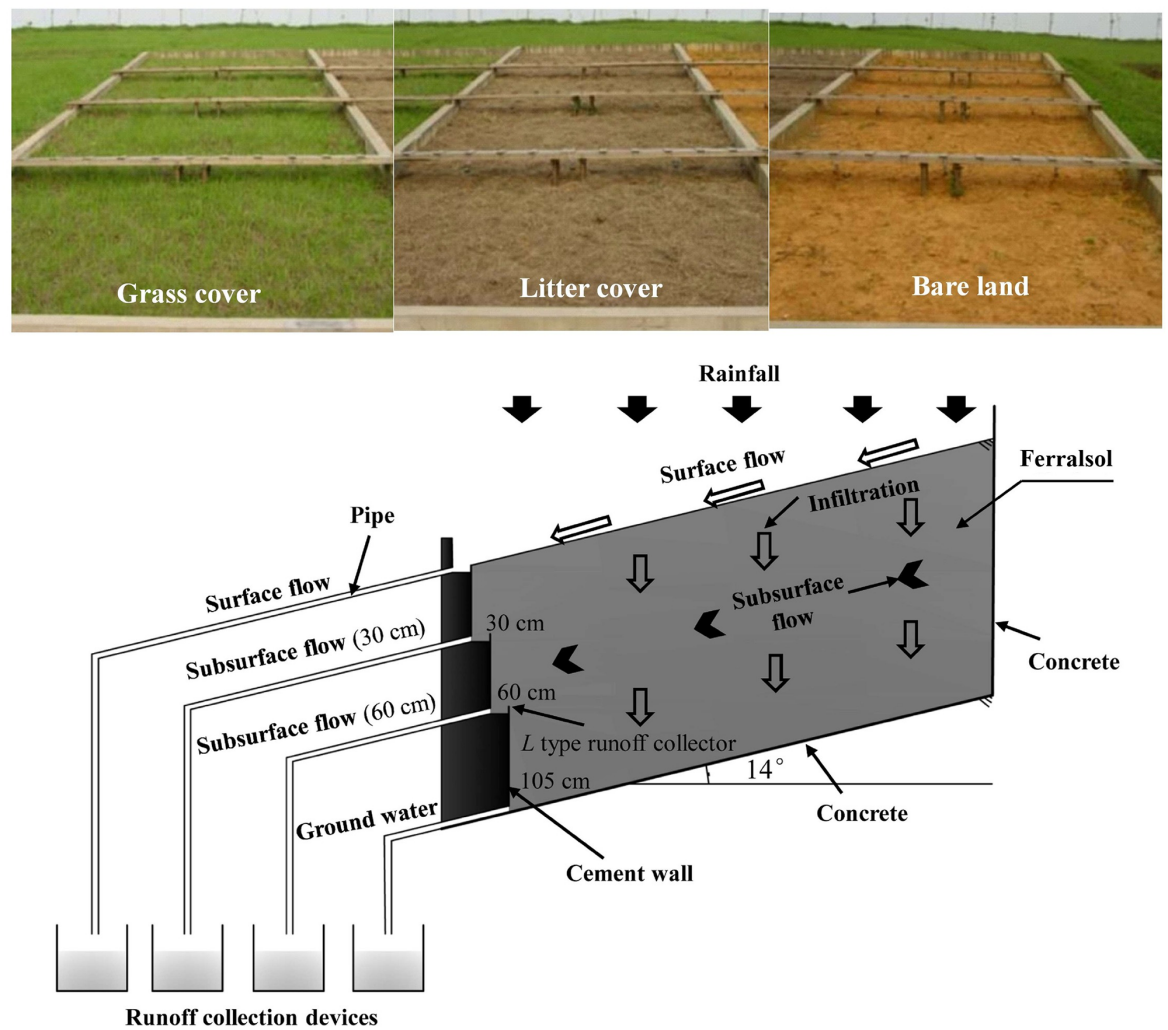
Schematic diagram of runoff generation in the three plots.

Given the low hill topography in the Ferralsol region, three runoff plots of 15 m×5 m×1.10 m (long×wide×deep) were established on broad hillslopes with the same conditions: a 14° slope and a southeast-facing aspect. Three plots were hydrologically isolated using cement walls to avoid lateral seepage from adjacent plots (Fig 1). Each runoff plot was packed with a 10-cm layer of gravel at the bottom and a 100-cm layer of test soil. The moisture content of the test soil was determined before packing the plots to calculate the soil volume. To ensure uniformity, the plots were packed in individual 10 cm layers. Before packing the upper soil, the soil surface was scratched using a 1-cm-thick board to prevent soil stratification.

Three types of surface cover were tested on the runoff plots (Fig 1). A grass cover plot was seeded at a high sowing density (20 g·m^-2^) with *Paspalum notatum*, a common grass species that supports soil and water conservation in the Ferralsol region. A litter cover plot was covered with a 5-cm-thick layer of *Paspalum notatum* cuttings that was maintained every three months during the study period. The grass and litter covered 100% of the plots. A bare land plot with no cover served as the control. The surface of the bare land plot contained no vegetation and was weeded manually every three months.

Three conflux troughs were built at the bottom of each plot to collect surface and subsurface flows (Fig 1). The surface and subsurface flows were then transferred from the conflux troughs to runoff storage containers using a pipe. The volumes of the storage containers used to collect the surface and subsurface flows were 3 m^3^ and 1 m^3^, respectively; flows were collected using the five-hole shun method. The surface flow conflux trough was set at the soil surface of each plot. Given the effects of the solum configuration and surface cover on subsurface flow, the subsurface flow conflux troughs were set at the bottom of the Ah layer (30 cm) and Bs layer (60 cm).

Automatic water stage recorders were installed in the storage containers to record surface and subsurface discharge at 5-min intervals. A meteorological station was located near the runoff plots to collect climate data. In this paper, precipitation was measured at 5-min intervals using a rain gauge. Runoff plot construction, grass planting and litter mulching were completed in 2000, and automatic and manual observations of runoff under natural rainfall conditions began in 2001.

### Statistical analysis

In this study, the k-means clustering method was chosen to distinguish between rainfall patterns using rainfall depth, maximum 30-min rainfall intensity and rainfall duration. Analysis of variance (ANOVA) was used to compare treatment means; the differences between the individual means were tested using the least significant difference multiple-comparison test. Statistical significance was evaluated at *P* < 0.05. The statistical procedures were conducted using the SPSS 17.0 software package for Windows.

Data from the years 2010 to 2012 were used in this study to analyze the characteristics of runoff in Ferralsols. The total runoff value (*TRV*), surface flow value (*SFV*) and subsurface flow value (*SSFV*_30_ and *SSFV*_60_) were calculated using the following formulas:

$$TRV=\;\frac{TRD}{P}\times 100\text{\%}$$ (1)

where *TRD* is the mean total runoff depth (mm) and *P* is the rainfall depth (mm),

$$TRD=SFD+SSFD_{30}+SSFD_{60}$$ (2)

$$SFV=\;\frac{SFD}{P}\times 100\text{\%}$$ (3)

$$SSFV_{30}=\;\frac{SSFD_{30}}{P}\times 100\text{\%}$$ (4)

$$\;SSFV_{60}=\;\frac{SSFD_{60}}{P}\times 100\text{\%}$$ (5)

where *SFD* is surface flow depth (mm), *SSFD*_30_ is subsurface flow depth (mm) at 30 cm, and *SSFD*_60_ is subsurface flow depth (mm) at 60 cm.

## Results

### Rainfall patterns

During 2010–2012, 114 natural rainfall events produced subsurface flows. These rainfall events were divided into four groups using k-means clustering according to rainfall duration, depth and maximum 30-min intensity (Table 1). Rainfall pattern I (RP I) was characterized by a long duration, great depth and low intensity and had an occurrence probability of 21.05%. Rainfall pattern II (RP II) had the longest duration (2632 min) and greatest depth (65.0 mm), as well as the lowest rainfall intensity (6.5 mm·h^-1^). Rainfall pattern II accounted for 5.26% of the rainfall events analyzed. Rainfall pattern III (RP III) was characterized by a short duration, shallow depth and high intensity. Rainfall pattern IV (RP IV) had the shortest duration (257 min), the shallowest depth (26.8 mm), and highest intensity (28.0 mm·h^-1^). Rainfall patterns III and IV accounted for 39.47% (45 events) and 34.21% (39 events) of the rainfall events studied, respectively.

**Table 1:** Eigenvalue statistical features of the four rainfall patterns.

Rainfall pattern	Rainfall duration (min)			Rainfall depth (mm)			*I*_30min_ (mm·h^-1^)		Number of events
	Sum	Mean	SD	Sum	Mean	SD	Mean	SD	
**RP I**	38729	1614	125	1388.1	60.4	8.5	12.3	7.4	24
**RP II**	15792	2632	278	389.8	65.0	10.3	6.5	2.0	6
**RP III**	41474	922	197	1263.8	28.1	9.1	9.1	3.9	45
**RP IV**	9838	257	134	1013.2	26.8	17.5	28.0	12.3	39

**Fig 2:**
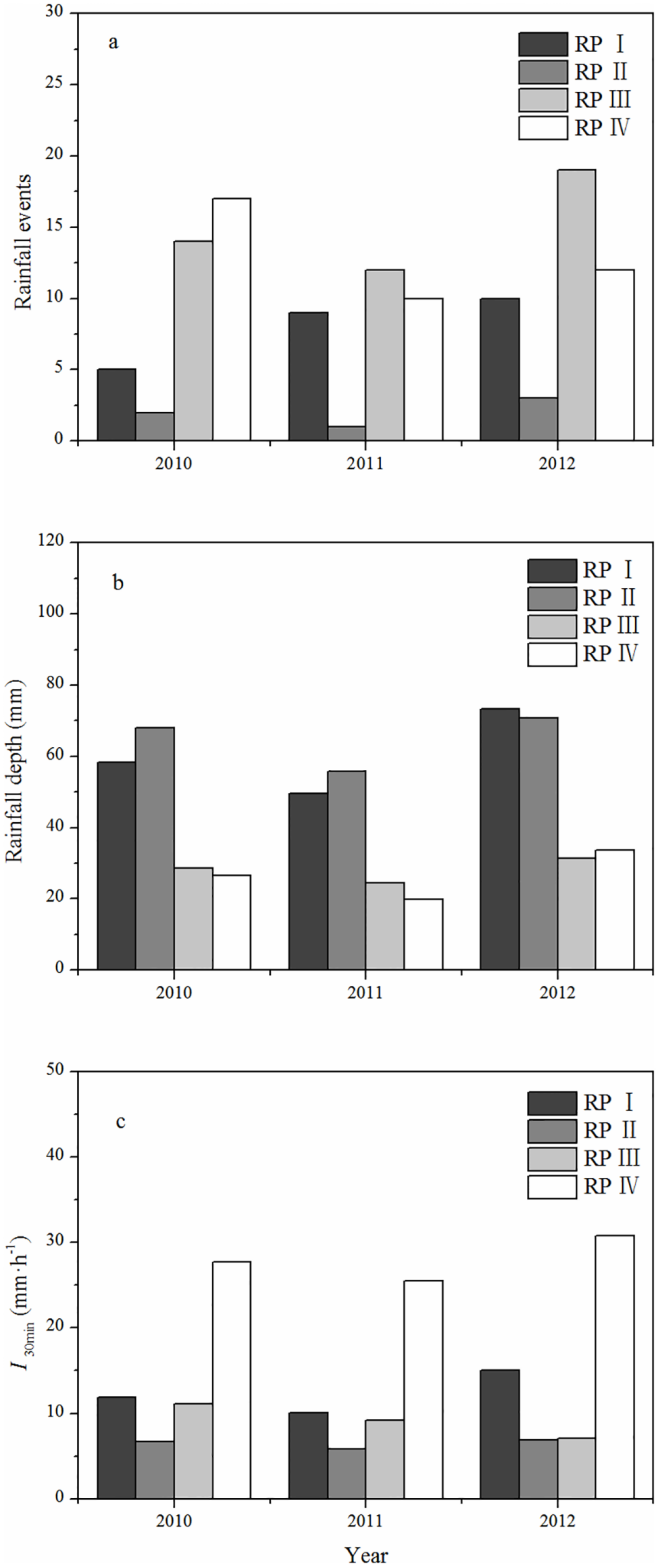
Rainfall pattern characteristics for events from 2010 to 2012.

**Fig 3:**
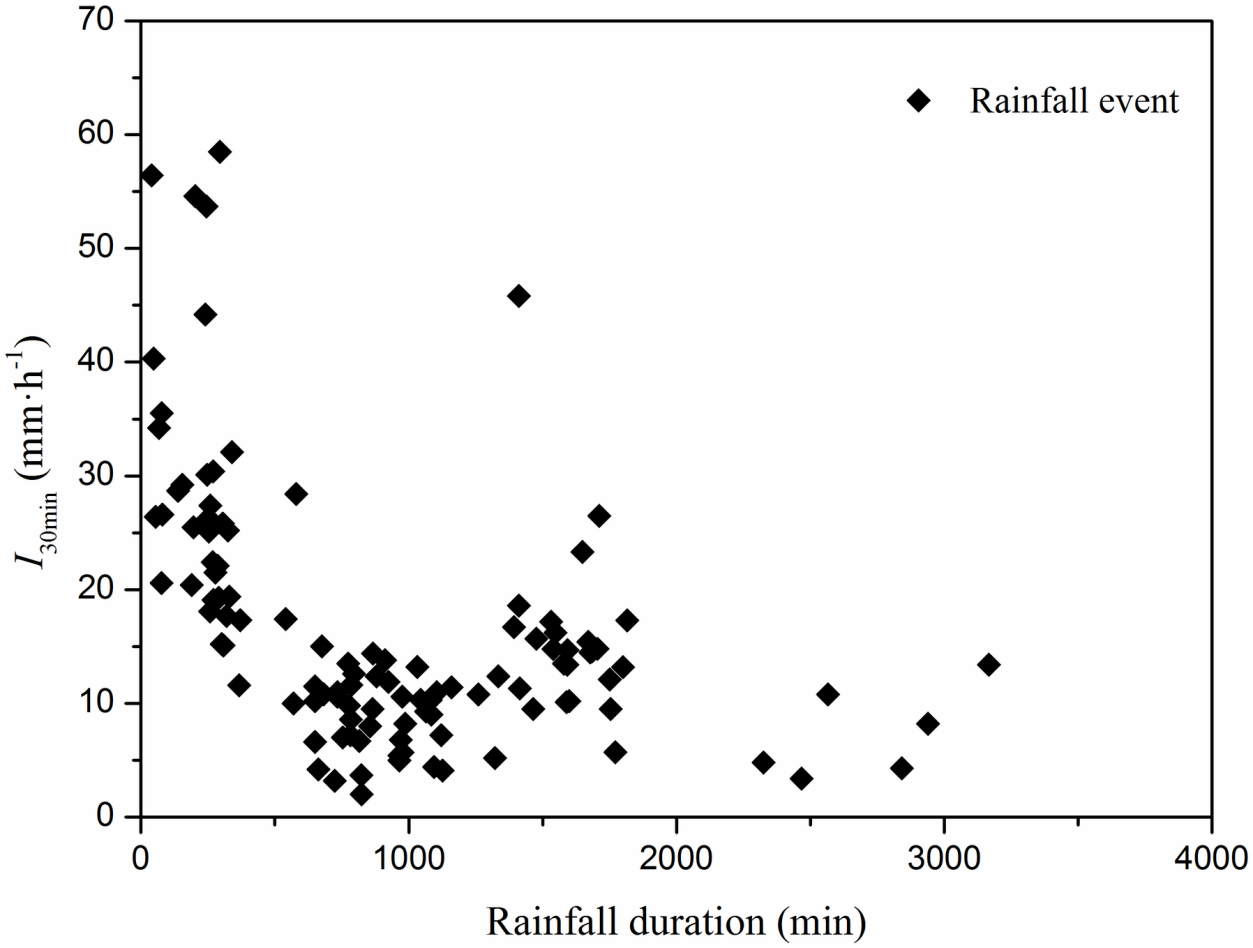
Relationship between maximum 30 min intensity and duration of the 144 rainfall events.

Fig 2 shows the duration, depth and intensity for the four rainfall patterns from 2010 to 2012. Fig 2(a) shows the number of events for each of the four rainfall patterns during the study period. The high-intensity, low-duration events were more common than the low-intensity, high-duration events. Fig 2(b) shows that rainfall patterns I and II had the greatest rainfall depth. The largest mean *I*_30min_ values were associated with rainfall pattern IV, as shown in Fig 2(c). The relationship between *I*_30min_ and rainfall duration for the 114 events is shown in Fig 3. Rainfall events with *I*_30min_ > 20 mm·h^-1^ rarely lasted long and were usually associated with rainfall durations of < 500 min.

### Total runoff

**Fig 4:**
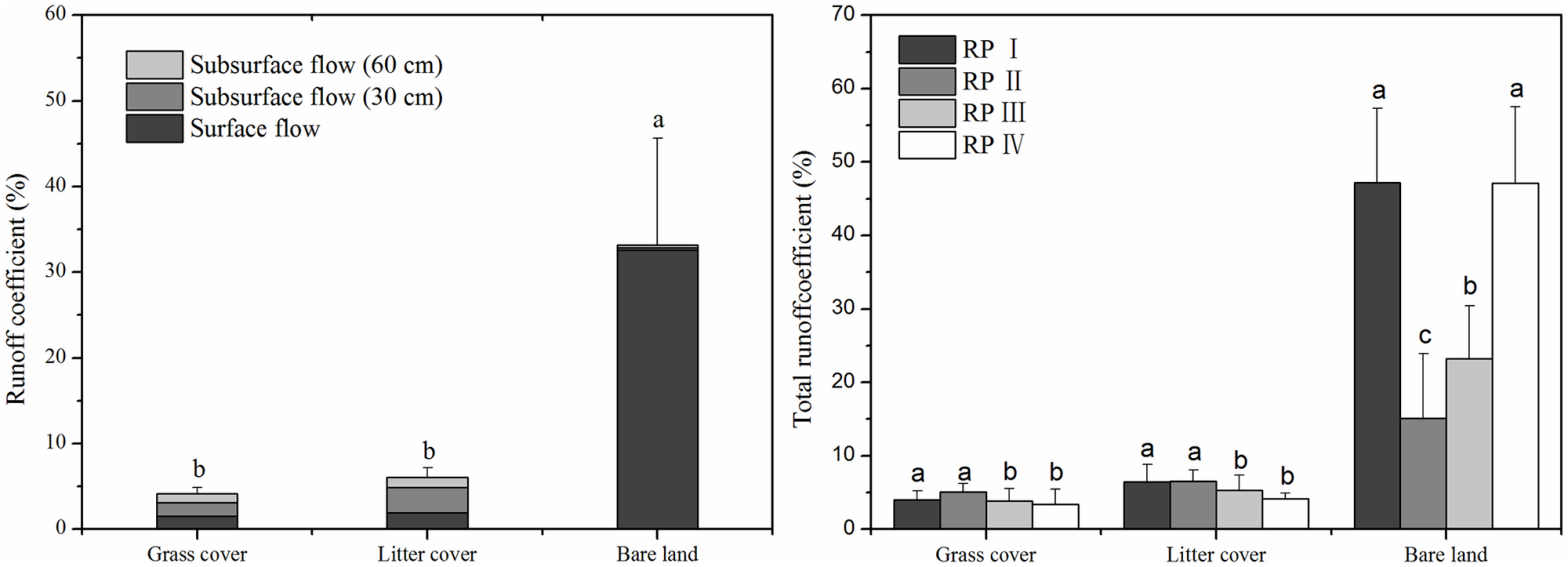
Total runoff values of the three land cover types under different rainfall patterns.

With respect to the runoff characteristics of the three land cover types at different layers (Fig 4), subsurface flow (30 cm) was the primary runoff component associated with the grass and litter cover types and accounted for 38.52%-48.93% of the events. However, surface flow was the primary runoff component associated with bare land, accounting for98.28% of events. Total runoff values for the 114 natural rainfall events can be arranged as follows: Bare land (33.15%) > litter cover (6.05%) > grass cover (4.18%). The total runoff value resulting from bare land was five to eight times greater than that from the grass and litter cover types, with no significant difference between the latter two.

The total runoff values associated with land cover under the four rainfall patterns are summarized in Fig 4. Under different rainfall patterns, the total runoff values for the grass and litter cover types were 3.37%-6.54%, while that of bare land was 15.13%-47.20%. Hence, the rainfall patterns relative to runoff could be ordered as follows: I and IV > III > II. Rainfall pattern IV exhibited the greatest total runoff when over bare land, which was three times greater than that of rainfall pattern II. However, the total runoff values related to the grass or litter cover types for the four rainfall patterns exhibited the following pattern: I and II > III and IV. Thus, total runoff increased as rainfall intensity increased for bare land and as rainfall depth increased for the grass and litter cover types. Moreover, total runoff can be reduced five- to eight-fold by covering the surface with grass or residues, regardless of rainfall pattern.

### Surface flow

**Fig 5:**
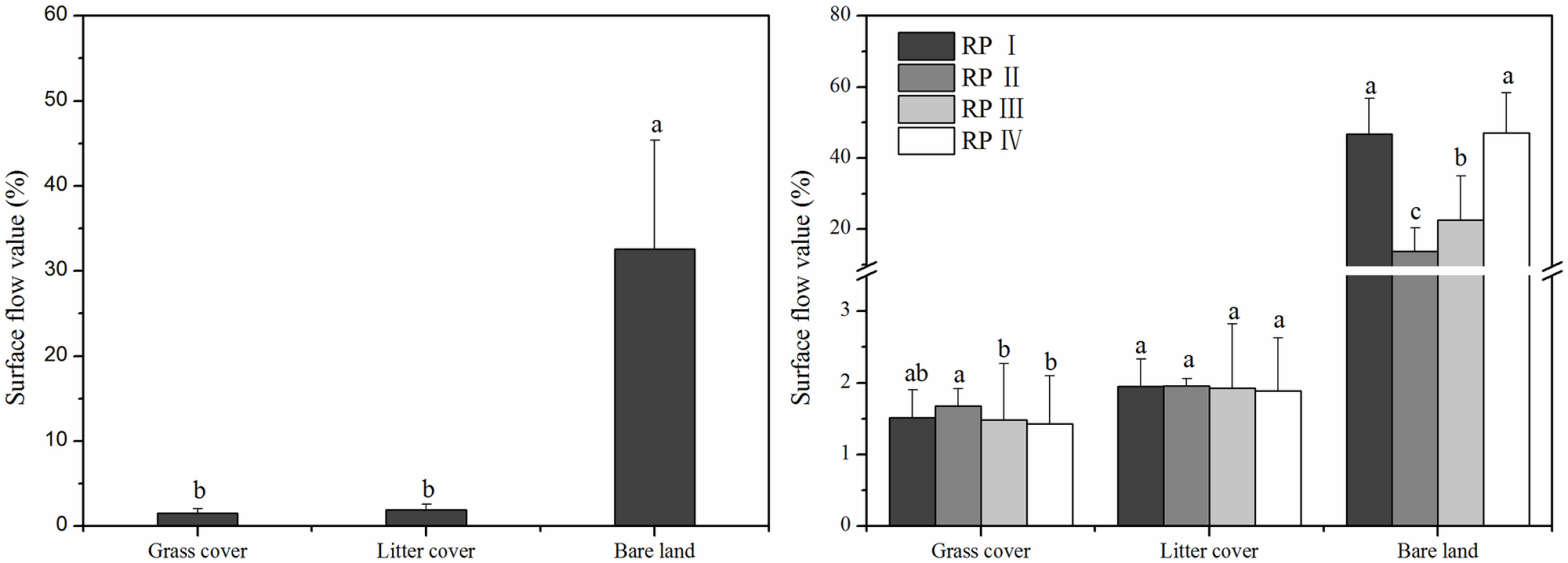
Surface flow values of the three land cover types under different rainfall patterns.

As shown in Fig 5, the ANOVA test and multiple comparisons revealed that the surface flow values from the grass cover (1.53%) and litter cover (1.93%) plots were similar, and both were significantly lower (*P* < 0.05) than those from the bare land plot (32.58%). Thus, using grass or residues to cover bare soil can clearly reduce surface flows to values eighteen to twenty-three times less than that of bare land.

The surface flow values for grass cover and litter cover exhibited no significant changes under different rainfall patterns and varied from 1.43%-1.96%, whereas the surface flow values for bare land ranged from 13.85%-47.04%. The bare land surface flows were on the same order as the total runoff. The largest surface flow values from the bare land plot under rainfall patterns I and IV were three times greater than the lowest values under rainfall patterns II and III. The surface flow values associated with the covered plots increased as rainfall depth increased but without significant differences. Therefore, using living vegetation or litter as land cover reduced the influence of rainfall pattern variations on surface flow.

### Subsurface flow

**Fig 6:**
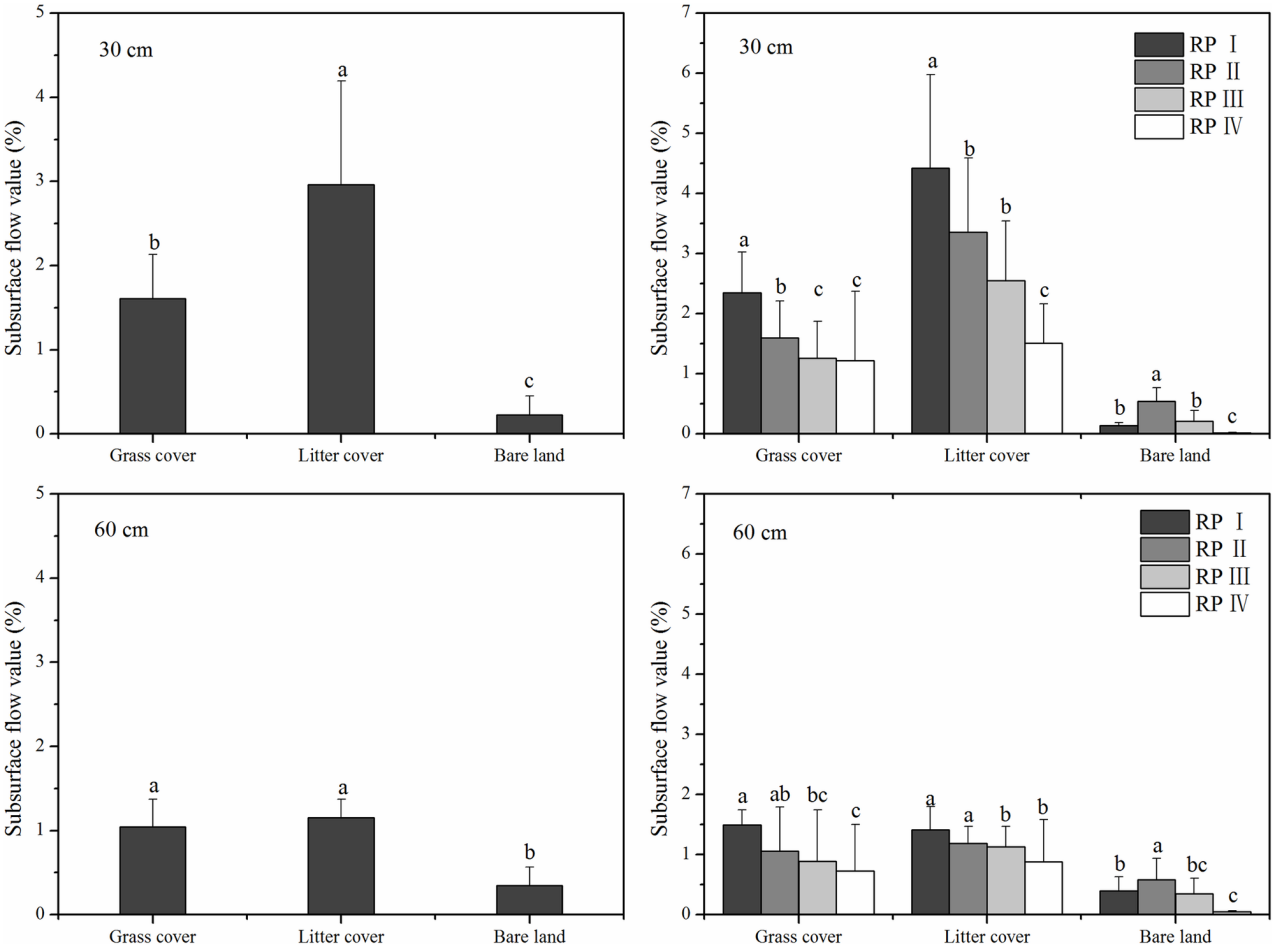
Subsurface flow values of three land cover types under different rainfall patterns.

The subsurface flow values of the three land cover types at depths of 30 and 60 cm are shown in Fig 6. The differences among the subsurface flows (30 cm) from the three land cover types reached a significant level, with the following order: litter cover (2.96%) > grass cover (1.61%) > bare land (0.23%). The grass cover and litter cover values were eleven to twenty-three times greater than the value found for bare land. The subsurface flow values (60 cm) for grass cover (1.04%) and litter cover (1.15%) were more similar to each other and nearly four times greater than those for bare land (0.35%). Therefore, subsurface flow characteristics for the three land cover types varied from the total runoff and surface flow characteristics.

As shown in Fig 6, the subsurface flow values for bare land under the four rainfall patterns were as follows: rainfall pattern II > rainfall pattern III > rainfall pattern I > rainfall pattern IV. Rainfall pattern II, which was characterized by low rainfall intensity, accumulated the most subsurface flows. However, rainfall pattern I, which was characterized by a high rainfall depth, created the most subsurface flows in the grass cover and litter cover plots. In contrast to the total runoff and surface flow values, rainfall pattern IV generated the least subsurface flow for bare land (0.02%-0.05%), whereas rainfall pattern II generated the highest value for bare land (0.54%-0.58%). Grass and litter cover clearly increase subsurface flow generation. For bare land, long-duration, low-intensity rainfall patterns can be beneficial to the generation of subsurface flows. The rainfall patterns had greater effects on subsurface flows than on total runoff and surface flows in the covered land types.

## Discussion

### Effects of different land cover types on subsurface flow generation

Various studies have reported that changes in land use or land cover impact storm runoff generation, resulting in changes to flood peak and volume [29–32]. As is shown in Figs 4–6, grass cover and litter cover were associated with lower surface flows and higher subsurface flows compared to bare land, with a consistently small proportion of precipitation forming overland flows. This conclusion is in accord with the results reported by Fullen et al. [33], Li et al. [34] and Zhang et al. [27], who showed that land cover changes lead to a decrease in total runoff by increasing the soil water storage capacity and infiltration.

The growth of grass roots creates a large number of stable channels. These root channels are conducive to preferential flow [35–37], which is an important constituent of subsurface flow. Mixing topsoil and litter can increase the hydraulic conductivity of the soil in the long term [38]. As shown in Table 2 [34], the topsoil’s (0–30 cm) physical properties (i.e., bulk density and soil porosity) changed over time for the thee land cover types studied. The permeability of topsoil with a root layer and incorporated litter (0–30 cm) was enhanced in the grass and litter cover plots. The increase in topsoil permeability provided favorable conditions for subsurface flow generation. Consequently, grass cover and litter cover significantly reduced surface flows and increased subsurface flow generation in the Ferralsol region, especially at a depth of 30 cm (Fig 6).

**Table 2:** Physical properties of topsoil (0~30 cm) for three land cover plots.

Land cover	Bulk density (g·cm^-3^)	Capillary porosity (%)	Total porosity (%)
**Grass cover**	1.19	41.70	53.30
**Litter cover**	1.25	40.11	51.17
**Bare land**	1.35	41.39	46.64

**Fig 7:**
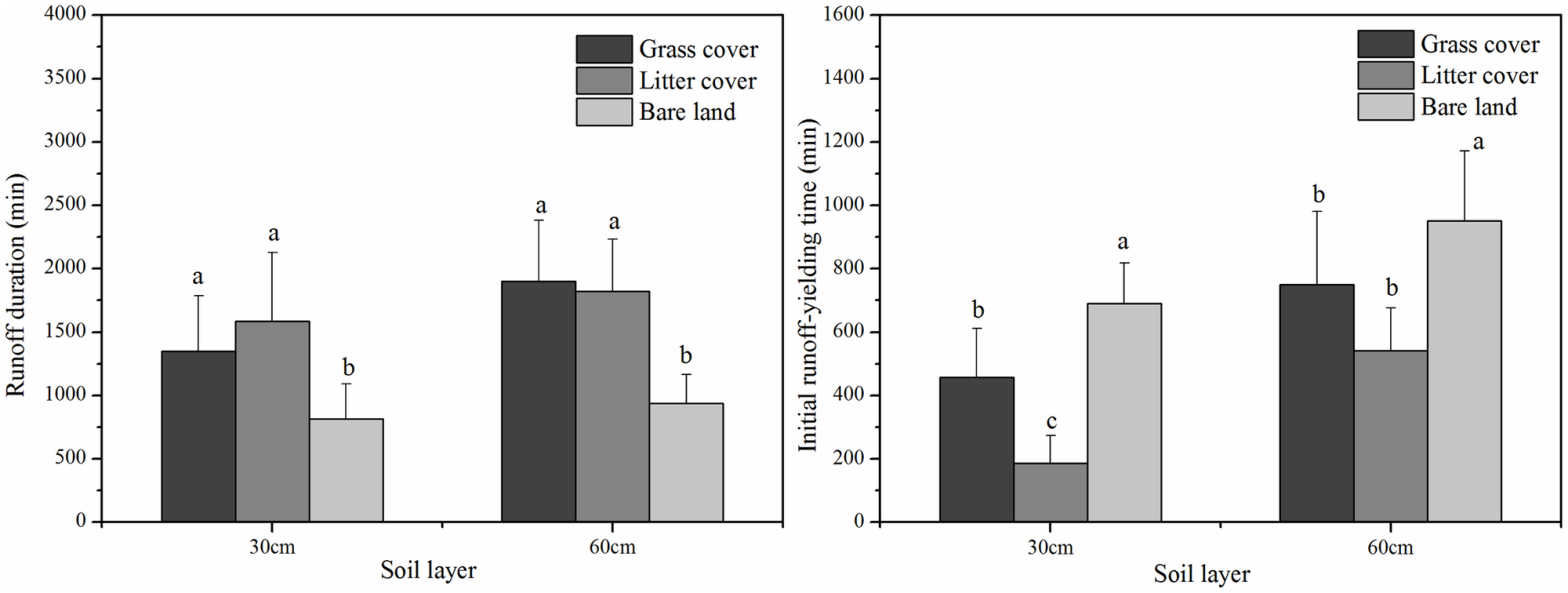
Duration and initial runoff yield time of subsurface flow generation under three land cover types.

Fig 7 shows that the duration of subsurface flows associated with bare land was less than that associated with the covered land types, but the situation was reversed with respect to initial runoff yield time. This result partially occurred because soil infiltration was higher in the covered plots than in the bare land plot. The presence of grass roots significantly reduced the quantity and energy of surface flows and increased topsoil infiltration and subsurface flows [37]. Moreover, Tang et al. [39] reported that dry antecedent moisture conditions promoted the occurrence of subsurface flows. The presence of a root layer in the grass cover plot led to an increase in soil evapotranspiration. The antecedent soil moisture was lower in the grass cover plot, resulting in a longer initial runoff yield time (Fig 7). Hence, the subsurface flow (30 cm) value for the grass cover plot was significantly less than that for the litter cover plot. However, no significant difference in initial runoff yield time for subsurface flows (60 cm) was observed between the grass cover and litter cover plots, likely because grass cover (*Paspalum notatum*) roots occurred between 0 and 30 cm. Therefore, grass and litter cover are excellent choices for decreasing surface flow and increasing subsurface flow, as they significantly reduce the response rate of subsurface flows to rainfall, especially at a depth of 30 cm.

### Effects of different rainfall patterns on subsurface flow generation

Table 1 shows that rainfall patterns I and II included storms of low intensity but high rainfall depth and long durations, particularly rainfall pattern II, which was characterized by the greatest depths and lowest intensities. This type of rainfall event mainly occurred from March to May. Rainfall patterns III and IV had characteristically short durations and little depth but high rainfall intensities; these storms primarily occurred in the summer and represented 73.68% of all rainfall events.

**Fig 8:**
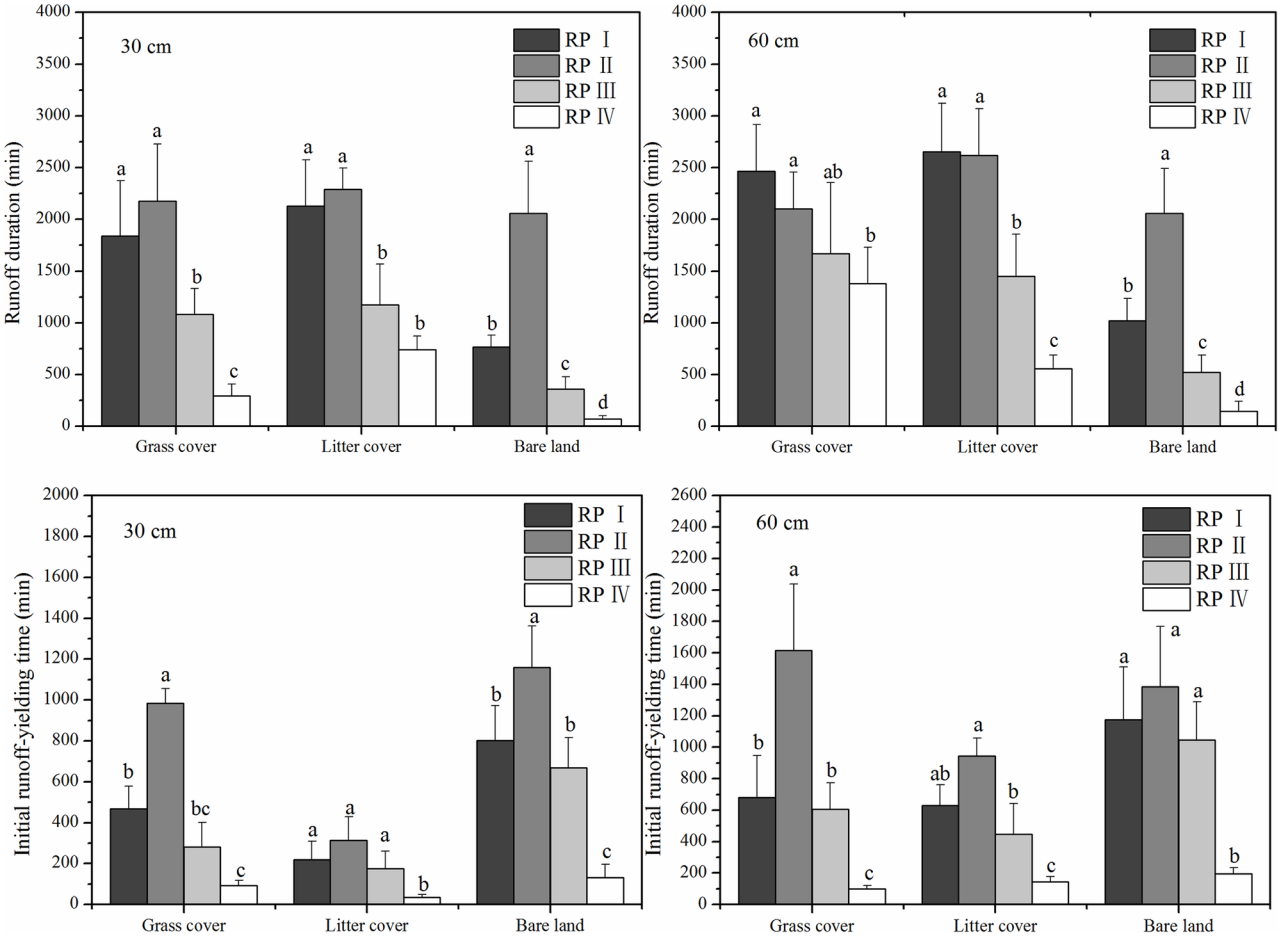
Runoff duration and initial runoff yield time for subsurface flow generation under different rainfall patterns.

The natural rainfall patterns studied clearly influenced the formation of runoff and the timing of flood peaks [40]. High precipitation, soil moisture content and near-surface hydraulic gradient values also impacted the surface-subsurface flow characteristics [41–43]. For the grass cover and litter cover plots, total runoff had a significant, positive relationship with rainfall depth (Table 3) and increased as rainfall depth increased (Fig 4). As shown in Fig 5, the subsurface flow values for rainfall patterns I and II, which were low-intensity and long-duration events (average mean of 4.22%) were higher than those for rainfall patterns III and IV, which were short-duration, high-intensity events (average value of 2.54%). Fig 8 shows that the subsurface flow duration and initial runoff yield time for rainfall patterns I and II (average values of 2015 min and 823 min) were greater than those for rainfall patterns III and IV (average values of 787 min and 369 min). These findings indicate that a prolonged, low-intensity rainfall pattern is much more likely to facilitate subsurface flows than a short, high-intensity rainfall event.

**Table 3:** Pearson correlations for runoff values and rainfall characteristics.

Runoff layer	Land cover	Rainfall duration	Rainfall depth	*I*_30min_
**Total runoff**	Grass cover	0.284	0.359*	0.204
	Litter cover	0.012	0.415*	-0.033
	Bare land	-0.184	0.134	0.553**
**Surface flow**	Grass cover	0.040	0.397*	0.213
	Litter cover	0.007	0.429*	0.165
	Bare land	-0.217	0.297	0.494**
**Subsurface flow (30 cm)**	Grass cover	0.289	0.518**	-0.443**
	Litter cover	0.255	0.339*	-0.156
	Bare land	-0.264	-0.415*	-0.387*
**Subsurface flow (60 cm)**	Grass cover	0.041	0.363*	-0.323*
	Litter cover	0.273	0.412*	0.038
	Bare land	0.291	0.268	-0.573**

*, Significant at P<0.05

**, Significant at P<0.01.

In the bare land plot, the largest surface flow values occurred under rainfall patterns I and IV and were three times greater than the lowest values under rainfall patterns II and III (Fig 5). Table 3 shows that the total runoff and surface flow values correlated significantly with rainfall intensity (*I*_30min_). Rainfall pattern IV had the highest rainfall intensity and highest surface flow values. However, the subsurface flow value for rainfall pattern IV (0.07%) was much lower than that for rainfall patterns I-III (0.54%, 1.12% and 0.56%, respectively). Additionally, the subsurface flow duration of rainfall pattern IV was the shortest, which may be a result of poor soil properties and the bare soil surface (Fig 8). In contrast, the low-intensity, long-duration rainfall pattern II generated the largest subsurface flow and longest runoff duration values. These statistical analyses also support the results above and are somewhat similar to those derived by Fu et al. [44] and Xie et al. [18]. Most of the rainwater infiltrated the soil, with subsurface flows generated before the soil was saturated, when the soil water infiltration rate was higher than the rainfall intensity. In other words, a prolonged, low-intensity rainfall pattern is much more likely to facilitate deep percolation and subsurface flows than a short-duration, high-intensity rainfall pattern, regardless of soil depth. The low-intensity and long-duration characteristics both resulted in greater subsurface flows, whereas short-duration events resulted in less subsurface flow. However, the interaction between surface flow and subsurface flow and their combined effects on soil erosion and nutrient losses are still unclear in the Ferralsol region, and additional investigations and studies are needed to further refine our understanding in the future.

## Conclusion

Between 2010 and 2012, 114 natural rainfall events produced subsurface flow. These rainfall events were divided into four groups according to rainfall duration, rainfall depth and maximum 30-min rainfall intensity using the k-means clustering method. Rainfall patterns I and II had low intensities but large rainfall depths and long durations, particularly rainfall pattern II, which had the greatest depth and lowest intensity. This type of rainfall event primarily occurred from March to May. Rainfall patterns III and IV had low durations and depths but high rainfall intensities and primarily occurred in the summer (representing 73.68% of all rainfall events).

The total runoff and surface flow values for the bare land plot studied were five to eight times greater and eighteen to twenty-three times greater than those for the grass cover and litter cover plots, respectively. Subsurface flows exhibited an opposing trend and were the primary runoff component for the grass cover and litter cover plots, accounting for 63.40%-67.93% of the runoff therein. In contrast, surface flows were the primary runoff component (98.28%) associated with the bare land plot. Therefore, total runoff and surface flows increase with decreasing land cover, while subsurface flows increase with increasing land cover.

The surface and subsurface flows of three land cover types differed significantly as the rainfall patterns varied. Rainfall patterns with low intensities and long durations generated more subsurface flow in the grass cover and litter cover plots, whereas rainfall patterns with high intensities and short durations generated more surface flow in the bare land plot. Rainfall pattern I had the highest subsurface flow values for the grass cover and litter cover plots. The highest surface flow value for the bare land plot occurred under rainfall pattern IV.

## Supporting information

S1 File Statistical data of field observation. (XLSX)
